# Prognostic Value of the Goutallier Scale for Paravertebral Muscle Atrophy in Predicting Disability and Pain Outcomes in Degenerative Lumbar Spinal Stenosis: A Longitudinal Cohort Study of 100 Patients

**DOI:** 10.3390/brainsci15070674

**Published:** 2025-06-23

**Authors:** Giuseppe Corazzelli, Sergio Corvino, Chiara Di Domenico, Federico Russo, Vincenzo Meglio, Settimio Leonetti, Valentina Pizzuti, Marco Santilli, Alessandro D’Elia, Francesco Ricciardi, Sergio Paolini, Raffaele de Falco, Oreste de Divitiis, Vincenzo Esposito, Gualtiero Innocenzi

**Affiliations:** 1 Neurosurgery Department, Santa Maria delle Grazie Hospital, ASL Napoli 2 Nord, 80078 Naples, Italy; raffaele.defalco@aslnapoli2nord.it; 2 Division of Neurosurgery, Department of Neurosciences, Reproductive and Odontostomatological Sciences, “Federico II” University, 80131 Naples, Italy; sercorvino@gmail.com (S.C.); didomenico.chiara.nch@gmail.com (C.D.D.); dr.federicorussonch@gmail.com (F.R.); dedivitiis.oreste@gmail.com (O.d.D.); 3 Department of Neurosurgery, AORN Sant’Anna e San Sebastiano, 81100 Caserta, Italy; vinmeglio790@gmail.com; 4 Department of Neurosurgery, IRCCS Neuromed, 86077 Pozzilli, Italy; settimioleonetti@gmail.com (S.L.); vale.pizzuti@hotmail.it (V.P.); deliaale@gmail.com (A.D.); fricciardi1@yahoo.it (F.R.); sergio.paolini@uniroma1.it (S.P.); vincenzo.esposito@uniroma1.it (V.E.); 5 Department of Neurology, IRCCS Neuromed, 86077 Pozzilli, Italy; fkt.santillimarco@gmail.com

**Keywords:** lumbar spinal stenosis, Goutallier classification system, paraspinal muscle atrophy, Oswestry Disability Index, visual analog scale, prognostic outcomes

## Abstract

**Background/Objectives:** Degenerative lumbar spinal stenosis (LSS) is a prevalent cause of disability in elderly populations, often treated with decompressive surgery. However, postoperative functional outcomes are variable and influenced by factors beyond neural compression alone. This study aimed to investigate the prognostic significance of the Goutallier Classification System (GS), a radiological index of paravertebral muscle fatty degeneration, in predicting long-term postoperative disability and pain in elderly patients undergoing decompression for LSS. **Methods:** A retrospective cohort study was conducted on 100 elderly patients who underwent primary lumbar decompression surgery for LSS between January 2020 and July 2022, with a minimum two-year follow-up. Patients were stratified according to their preoperative GS grades assessed via MRI. The Oswestry Disability Index (ODI) and Visual Analog Scale (VAS) for pain were collected preoperatively and at follow-up. Changes in the ODI and VAS (ΔODI and ΔVAS) were analyzed to evaluate associations between GS grades and functional outcomes. **Results:** Significant improvements in the ODI (from 41.0 ± 17.5 to 16.9 ± 8.2) and VAS (from 6.23 ± 2.52 to 3.75 ± 2.38) were observed postoperatively (*p* < 0.01). However, higher GS grades were associated with greater residual disability and pain at follow-up, as well as with smaller postoperative improvements in these scores (*p* < 0.01 for ODI; *p* = 0.01 for VAS). Gender differences were noted, with females predominating in higher GS grades. No significant differences in comorbidities or complication rates were identified across GS subgroups. **Conclusions:** Preoperative paravertebral muscle degeneration, as measured by the GS, emerged as a significant predictor of postoperative disability and pain in elderly LSS patients. Incorporating GS assessment into preoperative planning may refine surgical risk stratification and inform shared decision-making to optimize long-term functional recovery.

## 1. Introduction

Lumbar spinal stenosis (LSS) is among the most prevalent degenerative spinal disorders, representing a leading cause of disability and the most common indication for spinal surgery in elderly patients [1]. Characterized by progressive narrowing of the spinal canal, LSS can result in significant neural and vascular compression, leading to pain, limited mobility, and impaired quality of life [2]. While decompressive surgery is a standard treatment, the outcomes remain inconstant, with postoperative pain and residual disability enduring in determined patient subgroups [3].

Recent advancements in imaging and grading systems have facilitated a deeper understanding of factors contributing to surgical outcomes in LSS. The Goutallier Classification System (GS), initially developed to stratify fatty infiltration in the rotator cuff muscles [4,5], has been adapted for the assessment of paravertebral musculature [4,6]. Atrophy of these muscles, often suggestive of chronic degeneration [7], has been linked to worse clinical features, including increased disability and pain severity [8]. Despite its emergent adoption, the prognostic value of GS in predicting postoperative recovery in LSS patients has not been entirely recognized [9].

Previous studies have demonstrated significant correlations between GS grades [9], patient age [8], and radiological measures such as cross-sectional areas (CSAs) of paravertebral muscles [10]. However, the relationship between GS grades and patient-reported outcomes, such as the Oswestry Disability Index (ODI) and Visual Analog Scale (VAS) for pain, remains underexplored. Understanding this relationship could provide critical insights into preoperative planning and the stratification of surgical risk. Patients’ postoperative disability might impact surgical results, especially considering that the paravertebral muscle may not be preoperatively investigated.

This study aims to evaluate the prognostic significance of the Goutallier Classification System in predicting long-term postoperative disability and pain outcomes in elderly patients undergoing decompressive surgery for degenerative LSS. Through the analysis of a cohort of 100 LSS elderly patients stratified by GS grades and followed for years after decompressive surgery, this study aims to assess the gap in the literature and offer a more comprehensive understanding of the role of paravertebral muscle degeneration in surgical prognosis for LSS patients.

## 2. Materials and Methods

### 2.1. Selection Criteria

This retrospective single-center study included elderly patients diagnosed with degenerative lumbar spinal stenosis (LSS) who underwent primary lumbar decompression surgery between January 2020 and July 2022. Patients were followed up for two years to assess changes in the Oswestry Disability Index (ODI) [11] and Visual Analog Scale (VAS) scores, comparing follow-up with preoperative baseline values. Patients were stratified based on their Goutallier grade of paravertebral musculature [6].

Inclusion criteria were a clinical diagnosis of LSS with neurogenic claudication (<200 m), refractory pain lasting ≥ 6 months, and functional impairment; radiological confirmation of Schizas grade A4, B, C, or D stenosis on MRI, in accordance with the original classification, which subdivides grade A into A1 through A4; availability of preoperative and follow-up ODI and VAS scores; and availability of preoperative lumbar MRI suitable for paravertebral muscle evaluation. Patients with grades A1–A3, indicating no or minimal dural sac compression, were excluded [3].

Exclusion criteria were the presence of lumbar disk herniation as primary diagnosis [12,13]; scoliosis with a Cobb angle of >20° [14]; lumbar spinal instability demonstrated by flexion–extension radiographs [15]; a history of previous lumbar spine surgery (revision procedures); incomplete clinical or imaging data; and Goutallier grade 0, which was excluded from the analysis.

Baseline characteristics are summarized in Table 1. This study adhered to the Declaration of Helsinki and local ethical guidelines. As a retrospective study, formal ethical committee approval was waived, given that all patient data were anonymized and analyzed in compliance with institutional policies. However, informed consent for participation and data use was obtained from all individuals.

### 2.2. Clinical Parameters

The Oswestry Disability Index (ODI) questionnaire comprises 11 items assessing limitations in daily activities, with each section scored on a 0–5 scale, where 5 indicates the most significant disability. The total score is expressed as a percentage of daily disability [11]. The ODI has been extensively validated in spinal pathologies and shows high internal consistency, with Cronbach’s α values typically ranging from 0.85 to 0.90 across various language versions and patient cohorts, as reported in recent systematic reviews and a 2023 validation study in a lumbar surgery population (α ≈ 0.87; 95 % CI 0.86–0.88) [16]. It also demonstrates excellent test–retest reliability (ICC > 0.90) in multiple translated versions. Its sensitivity to clinical change and construct validity have made it a reference standard for quantifying disability. In addition, the patient’s age and BMI were evaluated at the time of diagnosis [17].

The Visual Analog Scale (VAS) measures back and leg pain intensity on a 0–10 scale, where 0 represents “no pain” and 10 indicates “worst possible pain.” Both ODI and VAS scores were evaluated preoperatively and postoperatively to assess changes in disability and pain levels among the enrolled patients. The Charlson Comorbidity Index (CCI) and Clavien-Dindo (CD) Score were included to evaluate comorbidity burden and postoperative complications, respectively. Postoperative complications were classified using the Clavien-Dindo grading system, which, despite some limitations in retrospective analyses, provides a standardized and objective framework for reporting surgical morbidity.

### 2.3. Radiological Parameters

**Figure 1:**
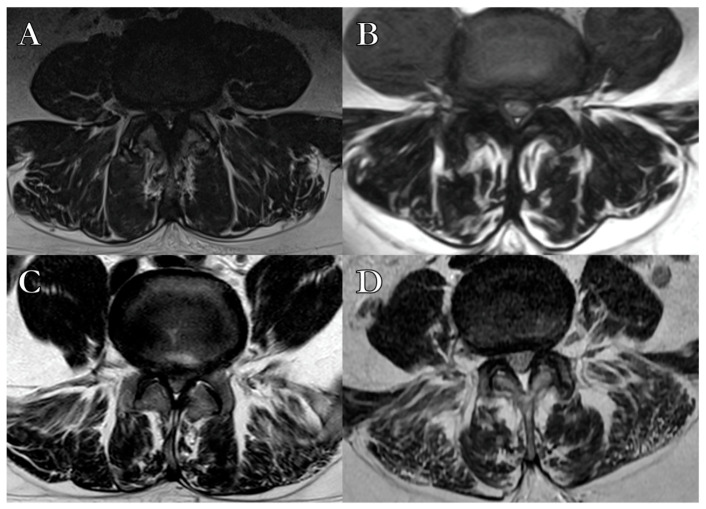
Semiquantitative classification of paralumbar muscles’ fatty degeneration according to the Goutallier Classification System. (**A**) Grade 1, few fatty streaks within the muscle. (**B**) Grade 2, Less than 50% fat within the muscle. (**C**) Grade 3, 50% fat within the muscle. (**D**) Grade 4, more than 50% fat within the muscle.

Preoperative lumbar/sacral MRI studies were performed with a 1.5 Tesla scanner, including STIR, T1-, and T2-weighted sagittal and axial images from L1 to S2. Axial images (4 mm slice thickness, 1 mm interslice gap) were centered at the intervertebral space parallel to the vertebral endplates. Images were analyzed using RadiAnt DICOM Viewer (Version 5). Fat infiltration of paravertebral muscles was preoperatively assessed on T2-weighted images, where hypointense muscle tissue and hyperintense adipose tissue were clearly distinguishable. Fatty degeneration was classified using the Goutallier system (GS) into four grades (Table 2): grade 0, no fat infiltration; grade 1, minimal focal or linear fat deposition; grade 2, fat deposition up to 50%; grade 3, fat deposition of 50%; and grade 4, fat deposition >50% (Figure 1) [18].

To assess interobserver agreement in Goutallier grade assignment, three independent authors independently reviewed the preoperative MRI scans. During the evaluation process, all three investigators were blinded to the patients’ clinical outcomes and treatment-related data to minimize potential bias in GS grade assignment. In cases of disagreement, a consensus was reached through discussion. If consensus could not be achieved, the final adjudication was performed by a senior author. While previous studies have employed quantitative methods to assess paravertebral muscle composition—such as CSA measurements or fat-to-muscle signal intensity ratios on advanced MRI protocols [19,20]—the present study adopted a validated semiquantitative visual inspection approach based on the Goutallier Classification System, as applied to T2-weighted axial images [6,10]. No quantitative measurement of muscle cross-sectional area or fat content was performed. This approach, although inherently subjective, has been validated in prior studies and enables routine application in standard imaging protocols without requiring additional sequences or software [2,6,8,9,15,18,21].

### 2.4. Surgical Technique

A conventional posterior lumbar laminectomy was performed on all enrolled patients. A midline posterior linear incision was made, followed by sharp dissection of the paravertebral muscles to the articular capsule without violating the capsule [22]. Complete removal of the spinous process and bilateral laminectomy to the articular processes was performed, followed by flavectomy and bilateral foraminotomy [13,22]. Adequate decompression was confirmed when the pulsating dura mater was free of osteoligamentous compression.

To address dural leaks, Gore-Tex 6–0 sutures, fibrin glue, and TachoSil were utilized. Hemostasis was achieved using Floseal and bipolar coagulation for muscle and venous plexus bleeding. No additional hemostatic agents were used. Anatomical layers were sutured with resorbable sutures [22].

### 2.5. Statistical Analysis

Preoperative and follow-up data were recorded and transcribed into Excel sheets (Version 16.92) and analyzed using GraphPad Prism (Version 10.3.1). Descriptive statistics were reported as means with standard deviations for continuous variables and as frequencies and percentages for categorical variables. The Shapiro–Wilk test was applied to assess the normality of distribution, with non-Gaussian variables defined by *p* < 0.05. Due to the limited sample size and the study design focusing on stratification by Goutallier grades, only non-adjusted bivariate analyses were performed.

Interobserver reliability was quantified using Cohen’s kappa (κ) statistic, calculated between pairs of raters prior to consensus resolution. The strength of agreement was interpreted according to standard benchmarks.

The Wilcoxon matched-pairs signed-rank test was employed to assess changes between preoperative and postoperative ODI and VAS scores within each Goutallier grade. The Friedman test was used to compare both absolute preoperative and postoperative scores and change scores (ΔODI and ΔVAS) across Goutallier grades. Statistical significance was set for *p*-value < 0.05.

## 3. Results

### 3.1. Sample Characteristics

A total of 168 elderly patients were initially identified. After excluding 68 patients due to missing follow-up data, the absence of preoperative MRI studies, or incomplete clinical records, 100 patients were ultimately included in the final analysis. The distribution of Goutallier grades was as follows: 9 patients in Grade I, 33 in Grade II, 36 in Grade III, and 22 in Grade IV. Interobserver agreement for Goutallier grade assignment was substantial, with a κ of 0.78. Patients were followed for a mean duration of 3.36 years (±0.68), with no significant differences in follow-up time across Goutallier grades (*p* = 0.14). To assess potential selection bias, baseline characteristics between included and excluded patients were compared. Variables analyzed included age, sex, the Charlson Comorbidity Index (CCI), Schizas grade, and preoperative ODI and VAS scores. Comparisons were performed using the Mann–Whitney U test for continuous variables and the chi-square or Fisher’s exact test for categorical variables, as appropriate. No statistically significant differences were observed between groups (*p* > 0.05 for all comparisons).

Preoperative and postoperative ODI and VAS scores were recorded and stratified by GS grade to evaluate the prognostic significance of this scale in predicting postoperative pain and disability in LSS patients.

The gender distribution revealed a predominance of males in the lower GS grades (100% in Grade I), with their proportion decreasing progressively to 18% in Grade IV. Conversely, the proportion of females increased with each grade, reaching 82% in Grade IV (*p* < 0.05). The mean age at surgery was 66.1 years (±7.3), ranging from 64.4 years (±6.42) in Grade I to 69.1 years (±8.32) in Grade IV, with no significant differences between GS grades (*p* = 0.39). Schizas grades and levels of stenosis were also comparable across the subgroups (*p* = 0.13 and *p* = 0.12, respectively), with Schizas Grade B (39%) and single-level stenosis (55%) being the most represented. The number of decompressed levels did not differ significantly across Goutallier grades (*p* = 0.12). Analysis of CCI and CD revealed no significant differences across GS grades (respectively, *p* = 0.74 and *p* = 0.36), indicating comparable comorbidity and postoperative complications (Table 2). A total of seven patients (7%) experienced intraoperative dural tears, all of which were successfully repaired intraoperatively using watertight suture techniques and adjuncts such as fibrin glue and TachoSil. No patients required reoperation for dural-related complications.

### 3.2. Clinical Outcomes

**Figure 2:**
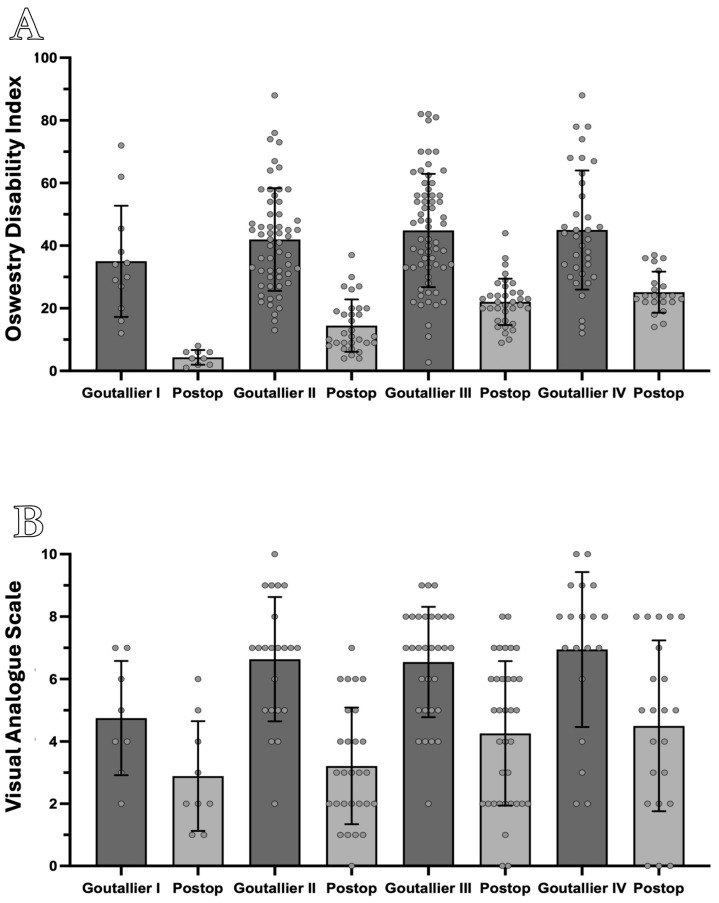
Preoperative and postoperative ODI and VAS scores stratified by Goutallier grade. (**A**) Preoperative and postoperative ODI scores stratified by Goutallier Classification show significant overall improvement, with better outcomes in lower grades and worse in advanced ones. (**B**) Preoperative and postoperative VAS scores demonstrate significant pain reduction, with lower postoperative pain in lower Goutallier grades and higher scores in advanced grades.

The mean preoperative ODI score was 41.0 (±17.5), improving significantly to 16.9 (±8.2) at follow-up (Wilcoxon matched-pairs signed-rank test, *p* < 0.01). When stratified by Goutallier grade, postoperative ODI scores were 4.33 (±2.35) for Grade I, 14.4 (±8.82) for Grade II, 22.1 (±7.39) for Grade III, and 25.1 (±6.56) for Grade IV (Figure 2A).

Similarly, the mean preoperative VAS score decreased from 6.23 (±2.52) to 3.75 (±2.38) postoperatively (Wilcoxon matched-pairs signed-rank test, *p* < 0.01). Postoperative VAS scores by Goutallier grade were 1.83 (±1.76) for Grade I, 3.21 (±1.87) for Grade II, 4.26 (±2.32) for Grade III, and 4.50 (±2.74) for Grade IV (Figure 2B).

Bars represent means ± standard deviations. Comparisons within grades were performed using the Wilcoxon matched-pairs signed-rank test; inter-grade comparisons of change scores were analyzed using the Friedman test.

**Figure 3:**
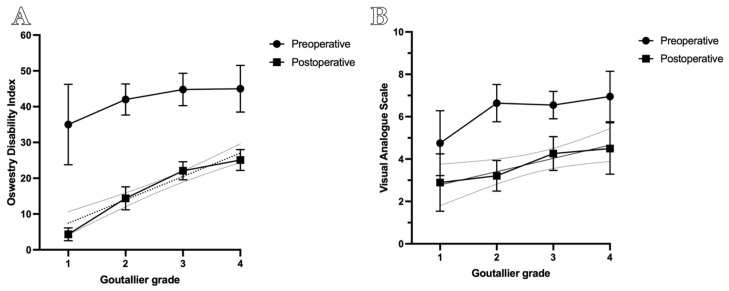
Correlation between Goutallier grade and postoperative ODI and VAS scores. (**A**) Postoperative ODI scores show a significant linear increase with higher Goutallier grades, reflecting greater residual disability in advanced paravertebral musculature degeneration (R = 0.39; *p* < 0.01). (**B**) Postoperative VAS scores exhibit a weaker yet significant trend of increased residual pain with higher Goutallier grades (R = 0.06; *p* = 0.01).

A significant trend was observed in postoperative ODI scores across Goutallier grades, with higher grades associated with greater residual disability (Friedman test: R = 0.39; *p* < 0.01). A weaker, yet statistically significant, trend was also noted for postoperative VAS scores (R = 0.06; *p* = 0.01), indicating a modest increase in residual pain with advancing Goutallier grade (Figure 3).

Scatterplots illustrate the linear trends observed across grades. Analysis was performed using the Friedman test.

Further analysis of change scores (ΔODI and ΔVAS) confirmed this pattern, demonstrating progressively smaller improvements in both disability and pain outcomes with increasing Goutallier grade. Detailed change scores are presented in Table 2.

## 4. Discussion

### 4.1. Preoperative Muscle Assessment as a Tool for Risk Stratification

This study estimated the prognostic utility of the GS in predicting long-term postoperative outcomes in elderly patients with degenerative LSS undergone decompressive surgery. For this purpose, 100 consecutive LSS-operated patients were followed for a mean of 3.36 years. The preoperative paravertebral muscles’ atrophy GS grades were assessed and prospectively monitored as four separate cohorts. Clinical postoperative data were retrospectively collected, recorded, and analyzed for statistical purposes.

The findings reveal significant improvements in both the ODI and VAS scores across all GS grades over the follow-up period. Conversely, higher GS grades were consistently associated with greater residual disability and pain. The linear trends observed in both curves highlight varying outcomes based on the degree of preoperative fatty degeneration in the paravertebral musculature. Furthermore, the analysis of change scores (ΔODI and ΔVAS) demonstrated that higher Goutallier grades were associated not only with greater residual disability and pain but also with smaller overall postoperative improvements, further underscoring the clinical impact of paraspinal muscle degeneration.

### 4.2. Impact of Paraspinal Degeneration on Disability and Pain Outcomes

The correlation between GS grades and postoperative ODI and VAS scores highlights the role of paravertebral muscle health in recovery. Previous studies have demonstrated that lower GS grades and greater lumbar muscle CSA values are associated with improved functional outcomes in LSS patients [2,8,10], although some have reported no significant association between the GS and ODI [8]. Our findings suggest that higher fatty infiltration of paravertebral muscles could impair functional recovery and pain resolution. Additionally, decreased paraspinal lean CSA has been associated with higher GS grades, reinforcing the relevance of muscle health in surgical prognosis [10]. Furthermore, higher GS grades have been linked to advanced age [7,23], decreased paraspinal CSA [2], and altered spinopelvic parameters, such as pelvic tilt and lumbar lordosis [21].

Beyond the impact of GS grades on postoperative outcomes, an intriguing observation in this study was the gender-related disparity in GS grades, with males predominating in the lower grades and females in the higher grades. A potential explanation for this lies in the role of estrogen and its association with visceral fat deposition [4]. Estrogen is recognized to promote fat storage, particularly in visceral and muscle compartments, which could contribute to hastened fatty degeneration of the paravertebral musculature in women [4]. This estrogenic influence on visceral obesity may explain the higher prevalence of advanced GS grades among female patients. Further research into hormonal and metabolic factors is warranted to clarify their contribution to paraspinal muscle health and their impact on surgical outcomes.

Paravertebral muscle atrophy has been studied to impact spinal balance and alignment [24], contributing to altered lumbar lordosis, thoracic kyphosis, and sacral–vertebral angles [21]. The interaction between muscle degeneration and spinal deformity emphasizes the importance of paravertebral muscle integrity in maintaining functional spinal alignment [21,25,26,27].

Studied as a foremost stabilizing muscle, the inverse relationship between Erector Spinae (ES) CSA and preoperative ODI values, as previously reported [28,29], underscores the role of muscle degeneration in patient-reported disability [10]. Our study builds on this by integrating GS grading, providing a broader context for assessing paraspinal muscle quality. Interestingly, despite its stabilizing role, ES-CSA showed no significant correlation with the dural-sac CSA, suggesting that functional impairment may not exclusively depend on canal width, but also on concurrent intrinsic muscle degeneration [29].

### 4.3. Comparison with Existing Literature and Pathophysiological Considerations

Previous studies have demonstrated that greater preoperative fatty infiltration of paraspinal muscles is associated with poorer postoperative outcomes, including persistent low back pain and diminished clinical improvement [23,30]. This investigation corroborates these findings, further emphasizing the critical role of paravertebral muscle quality in surgical prognosis. The consistent relationship between fatty infiltration, reduced cross-sectional areas of key muscles such as the multifidus and erector spinae, and heightened pain and functional impairment highlights the necessity of incorporating muscle quality assessment into preoperative planning. The absence of significant differences in CCI and CD across GS grades suggests that muscle degeneration, as measured by GS, supports the hypothesis that the GS independently influences postoperative disability and pain. Future studies should explore the interplay between GS, frailty indices, and functional recovery in larger, multicenter cohorts. However, our results contribute to the growing body of evidence, underscoring paraspinal fatty atrophy as a significant determinant of pain and disability.

Taken together, these findings underscore the critical need for a comprehensive approach to preoperative evaluation that considers both muscle degeneration and patient-specific characteristics. This study underscores the importance of incorporating the GS into preoperative evaluations for LSS patients. Identifying advanced muscle degeneration can aid in risk stratification, tailored interventions, and prehabilitation to enhance outcomes. Furthermore, the GS offers further elements for transparent preoperative discussions, assisting clinicians to manage expectations and support informed decision-making effectively. Although the present study did not investigate therapeutic interventions targeting paraspinal muscle quality, evidence from the rehabilitation and resistance training literature suggests that targeted physical therapy programs might reduce fatty infiltration and improve disability scores. However, randomized controlled trials confirming this hypothesis are lacking.

### 4.4. Advantages and Limits of a Semiquantitative MRI-Based Approach

Recent research addressed the GS as a reliable, reproducible and easy-to-use visual index for preoperative assessment of paravertebral musculature atrophy [6]. In this setting, paravertebral sarcopenia appears as a potential predictive factor for postoperative pain and subjective disability. The recent literature has increasingly embraced quantitative imaging techniques—such as muscle CSA calculations and signal-intensity mapping—to assess paraspinal muscle composition in spinal disorders [6,8,10,19,20]. While such methods undoubtedly offer higher resolution and objectivity, their integration into routine clinical practice remains limited by the need for dedicated sequences and post-processing software. In contrast, the Goutallier Classification System, despite its semiquantitative and visual nature, offers a pragmatic and reproducible tool applicable to standard MRI scans, with growing validation in the lumbar spine setting [6]. Our study deliberately adopted this approach, prioritizing feasibility and external reproducibility over precision metrics. Nonetheless, we acknowledge the limitations of this method and advocate for future studies to explore hybrid models that combine Goutallier grading with quantitative imaging indices to refine risk stratification.

### 4.5. Study Limitations and Interpretative Boundaries

This study has several limitations. The retrospective design may introduce selection bias, despite prospective follow-up. The single-center setting could affect generalizability. Additionally, reliance on conventional imaging may not fully capture the complexity of paraspinal muscle degeneration; advanced MRI techniques could enhance precision [23]. The impact of prehabilitation and rehabilitation was not assessed and warrants further investigation. Furthermore, the relatively small sample size within certain Goutallier subgroups, particularly Grade I, may have limited the statistical power to detect subtle differences and could impact the generalizability of the findings. Consequently, observed trends should be interpreted with caution, and validation through larger, multicenter studies is warranted. Due to the retrospective nature of the study, data regarding patients’ physical activity levels and adherence to postoperative rehabilitation protocols were not systematically recorded. This lack of standardization may have introduced variability in outcomes and represents an important limitation in interpreting the role of paravertebral muscle degeneration in functional recovery. While these factors could potentially influence paraspinal muscle conditions and outcomes, their assessment was beyond the scope and feasibility of this investigation.

Despite these limitations, our findings underscore the prognostic utility of the GS in lumbar spinal stenosis surgery. Future multicenter studies integrating comprehensive frailty assessments and standardized imaging protocols are needed to validate these results. Furthermore, integrating the Goutallier scale assessment with advanced quantitative imaging methods, such as Dixon MRI or chemical-shift imaging [31,32], could offer a more precise evaluation of muscle composition, particularly the fat-to-muscle ratio and water content, which may reveal subtle changes in paraspinal muscle integrity beyond the visual assessment of the GS [33,34,35]. These advanced imaging modalities can also help in understanding the interplay between muscle quality and global spinal balance, including sagittal alignment and spinopelvic parameters, which have been linked to postoperative outcomes in LSS [36]. By adopting such comprehensive imaging strategies, clinicians can refine preoperative risk stratification and potentially identify early indicators of sarcopenia or frailty-related complications, which are particularly relevant in elderly patients. Additionally, combining GS grades with patient-reported measures of physical activity and nutritional status may provide a more holistic evaluation of the factors influencing recovery trajectories after lumbar decompression. Such integrative approaches underscore the multifactorial nature of postoperative disability and highlight the need for personalized perioperative management pathways in the treatment of lumbar spinal stenosis.

### 4.6. Future Perspectives and Clinical Integration

Building upon these findings, future prospective studies should investigate whether targeted interventions aimed at preserving or restoring paraspinal muscle quality, such as structured rehabilitation programs, nutritional support, or hormonal modulation, might mitigate the adverse impact of muscle degeneration on postoperative recovery. Furthermore, integrating GS assessment with quantitative imaging methods and patient-specific frailty metrics may offer a more comprehensive stratification model. Future studies employing multivariate regression analyses may further elucidate the independent contribution of paravertebral muscle degeneration to functional outcomes, accounting for other relevant clinical and demographic variables. This approach may refine surgical prognostication and help personalize perioperative management. These directions may ultimately lead to personalized prehabilitation protocols capable of improving surgical outcomes in elderly patients with lumbar spinal stenosis.

In summary, the findings of the present investigation underscore the pivotal prognostic value of paravertebral muscle degeneration, as assessed by the Goutallier scale, in determining long-term functional and pain-related outcomes in elderly patients undergoing decompressive lumbar surgery for degenerative spinal stenosis. The discernible gradient of residual disability and pain severity across the spectrum of GS grades emphasizes the clinical utility of this straightforward yet robust radiological index. Beyond its traditional application, the Goutallier scale emerges as an essential adjunct in preoperative risk stratification, offering an additional layer of insight that complements conventional radiological and clinical evaluations. These observations collectively advocate for a paradigm shift in preoperative assessment strategies, underscoring the necessity of integrating muscle quality and broader frailty indices into individualized patient-centered care pathways. In this context, the incorporation of GS grading into routine clinical practice may ultimately enhance shared decision-making, refine surgical planning, and support tailored perioperative management strategies aimed at optimizing postoperative recovery and long-term functional independence.

## 5. Conclusions

This study provides evidence supporting the prognostic value of the GS in elderly patients undergoing decompressive surgery for degenerative lumbar spinal stenosis. Higher GS grades were associated with greater residual disability and pain, as well as with reduced postoperative functional gains, underscoring the relevance of paraspinal muscle quality in clinical outcomes.

While acknowledging the limitations inherent to the retrospective design, single-center setting, and lack of systematic assessment of rehabilitation protocols and preoperative activity levels, these findings suggest that the GS may serve as a valuable adjunct in preoperative risk stratification and patient counseling.

Further prospective, multicenter studies integrating standardized imaging assessments and frailty measures are needed to validate these results and to explore whether targeted interventions on paraspinal muscle health could improve surgical outcomes.

## Figures and Tables

**Table 1:** Goutallier Classification System grades.

Goutallier Classification System Grade	Percentage of Muscular Fatty Degeneration
0	No fatty infiltration
1	Few fatty streaks within the muscle
2	Less than 50% fat within the muscle
3	50% fat within the muscle
4	More than 50% fat within the muscle

**Table 2:** Baseline sample characteristics, stratified by Goutallier scale grades. Descriptive and inferential statistical analyses.

Goutallier Grade	Sample (*n* = 100)	Grade I (*n* = 9)	Grade II (*n* = 33)	Grade III (*n* = 36)	Grade IV (*n* = 22)	Statistical Test
**Gender ^†^**						
**Male**	48	9 (100)	21 (64)	14 (39)	4 (18)	
**Female**	52	0	12 (36)	22 (61)	18 (82)	***p* < 0.05**
**Age at surgery (yrs) ^‡^**	66.1 (±7.3)	64.39 (±6.42)	67.80 (±5.51)	67.47 (±7.97)	69.11 (±8.32)	*p* = 0.39
**Schizas grade ^†^**						
**A4**	16	1 (11)	10 (30)	2 (6)	3 (14)	
**B**	31	5 (56)	13 (39)	8 (22)	5 (23)	
**C**	40	2 (22)	9 (27)	21 (58)	8 (36)	
**D**	13	1 (11)	1 (3)	5 (14)	6 (27)	*p* = 0.13
**Levels of stenosis ^†^**						
**Single-level**	54	6 (67)	21 (64)	18 (50)	12 (41)	
**Two-level**	34	3 (33)	10 (30)	14 (39)	7 (32)	
**Three-level**	12	0 (0)	2 (6)	4 (11)	6 (27)	*p* = 0.12
**Preop CCI**	3.93 (±1.06)	4.1 (±0.96)	3.96 (±1.42)	3.7 (±1.35)	3.96 (±1.07)	*p* = 0.74
**Oswestry Disability Index ^‡^**						
**Preoperative**	41.0 (±17.5)	35.0 (±17.7)	42.0 (±16.4)	44.8 (±18.1)	45.0 (±19)	
**Follow-up**	16.9 (±8.2)	4.33 (±2.35)	14.4 (±8.82)	22.1 (±7.39)	25.1 (±6.56)	
**Wilcoxon matched test**		***p* < 0.01**	***p* < 0.01**	***p* < 0.01**	***p* < 0.01**	
**ΔODI**		30.67 (±16.65)	27.60 (±14.22)	22.70 (±15.76)	19.90 (±16.72)	Friedman test
						R = 0.39; ***p***** < 0.01**
**Visual Analog Scale ^‡^**						
**Preoperative**	6.23 (±2.52)	4.75 (±2.89)	6.64 (±1.99)	6.55 (±1.77)	6.95 (±2.48)	
**Follow-up**	3.75 (±2.38)	1.83 (±1.76)	3.21 (±1.87)	4.26 (±2.32)	4.50 (±2.74)	
**Wilcoxon matched test**		***p* = 0.01**	***p* = 0.02**	***p* < 0.01**	***p* = 0.01**	
**ΔVAS**		2.92 (±2.52)	3.43 (±1.93)	2.29 (±2.10)	2.45 (±2.62)	Friedman test
						R = 0.06; ***p***** = 0.01**
**CD Score**	1.88 (±0.92)	1.66 (±0.64)	2.17 (±1.13)	1.79 (±0.94)	1.98 (±1.2)	*p* = 0.36
**Mean follow-up (yrs) ^‡^**	3.36 (±0.68)	3.46 (±0.77)	3.48 (±0.69)	3.31 (±0.59)	3.13 (±0.49)	*p* = 0.14

CCI: Charlson Comorbidity Index; CD: Clavien-Dindo Score for postoperative complications. ^†^ Categorical variables are expressed as raw frequencies (percentages) and analyzed using the chi-square test. ^‡^ Quantitative variables are expressed as means (±standard deviations) and analyzed using the Wilcoxon matched-pairs signed-rank test for preoperative and follow-up comparisons within each cohort, and the Friedman test for comparisons between cohorts.

## Data Availability

The data supporting the reported results of this study are not publicly available due to privacy and ethical restrictions related to patient confidentiality. However, de-identified data may be made available from the corresponding author upon reasonable request and with the approval of the local ethics committee.

## Abbreviations

The following abbreviations are used in this manuscript:

LSS	Lumbar Spinal Stenosis
GS	Goutallier Classification System
ODI	Oswestry Disability Index
VAS	Visual Analog Scale
MRI	Magnetic Resonance Imaging
CSA	Cross-Sectional Area
CCI	Charlson Comorbidity Index
CD	Clavien-Dindo Classification
ΔODI	Change in Oswestry Disability Index
ΔVAS	Change in Visual Analog Scale

## References

[1] Steurer J., Nydegger A., Held U., Brunner F., Hodler J., Porchet F., Min K., Mannion A.F., Michel B. (2010). LumbSten: The lumbar spinal stenosis outcome study. BMC Musculoskelet. Disord..

[2] Mandelli F., Nüesch C., Zhang Y., Halbeisen F., Schären S., Mündermann A., Netzer C. (2021). Assessing fatty infiltration of paraspinal muscles in patients with lumbar spinal stenosis: Goutallier classification and quantitative MRI measurements. Front. Neurol..

[3] Schizas C., Theumann N., Burn A., Tansey R., Wardlaw D., Smith F.W., Kulik G. (2010). Qualitative grading of severity of lumbar spinal stenosis based on the morphology of the dural sac on magnetic resonance images. Spine.

[4] Slabaugh M.A., Friel N.A., Karas V., Romeo A.A., Verma N.N., Cole B.J. (2012). Interobserver and intraobserver reliability of the Goutallier classification using magnetic resonance imaging: Proposal of a simplified classification system to increase reliability. Am. J. Sports Med..

[5] Wall L.B., Teefey S.A., Middleton W.D., Dahiya N., Steger-May K., Kim H.M., Wessell D., Yamaguchi K. (2012). Diagnostic performance and reliability of ultrasonography for fatty degeneration of the rotator cuff muscles. J. Bone Jt. Surg. Am. Vol..

[6] Corazzelli G., Meglio V., Corvino S., Leonetti S., Ricciardi F., D’Elia A., Pizzuti V., Santilli M., Innocenzi G. (2024). The Goutallier Classification System: How does Paravertebral Adipose Degeneration Change in Patients with Symptomatic Lumbar Spinal Stenosis?. Spine.

[7] Tamai K., Chen J., Stone M., Arakelyan A., Paholpak P., Nakamura H., Buser Z., Wang J.C. (2018). The evaluation of lumbar paraspinal muscle quantity and quality using the Goutallier classification and lumbar indentation value. Eur. Spine J..

[8] Banitalebi H., Aaen J., Storheim K., Negård A., Myklebust T.Å., Grotle M., Hellum C., Espeland A., Anvar M., Indrekvam K. (2022). A novel MRI index for paraspinal muscle fatty infiltration: Reliability and relation to pain and disability in lumbar spinal stenosis: Results from a multicentre study. Eur. Radiol. Exp..

[9] Battaglia P.J., Maeda Y., Welk A., Hough B., Kettner N. (2014). Reliability of the Goutallier classification in quantifying muscle fatty degeneration in the lumbar multifidus using magnetic resonance imaging. J. Manip. Physiol. Ther..

[10] Corazzelli G., Capece M., Meglio V., Leonetti S., Pizzuti V., Ricciardi F., D’Elia A., Santilli M., Innocenzi G. (2023). Multiple univariate analysis of radiologic and clinical features on 168 patients with lumbar spinal stenosis: What is the role of the erector spinae in the development of a patient’s disability?. Acta Neurochir..

[11] Mehra A., Baker D., Disney S., Pynsent P. (2008). Oswestry Disability Index scoring made easy. Ann. R. Coll. Surg. Engl..

[12] Ding J.-Z., Kong C., Li X.-Y., Sun X.-Y., Lu S.-B., Zhao G.-G. (2022). Different degeneration patterns of paraspinal muscles in degenerative lumbar diseases: A MRI analysis of 154 patients. Eur. Spine J..

[13] Corazzelli G., Corvino S., Ricciardi F., Pizzuti V., Leonetti S., D’Elia A., Santilli M., Aloj F., Innocenzi G. (2024). Perioperative management of antithrombotic therapy in elderly patients undergoing lumbar discectomy: A retrospective study on 163 patients. Neurosurg. Rev..

[14] Korovessis P.G., Stamatakis M.V. (1996). Prediction of scoliotic Cobb angle with the use of the scoliometer. Spine.

[15] Bumann H., Nüesch C., Loske S., Byrnes S.K., Kovacs B., Janssen R., Schären S., Mündermann A., Netzer C. (2020). Severity of degenerative lumbar spinal stenosis affects pelvic rigidity during walking. Spine J..

[16] Koivunen K., Widbom-Kolhanen S., Pernaa K., Arokoski J., Saltychev M. (2024). Reliability and validity of Oswestry Disability Index among patients undergoing lumbar spinal surgery. BMC Surg..

[17] Davidson M., Keating J.L. (2002). A comparison of five low back disability questionnaires: Reliability and responsiveness. Phys. Ther..

[18] Kjaer P., Bendix T., Sorensen J.S., Korsholm L., Leboeuf-Yde C. (2007). Are MRI-defined fat infiltrations in the multifidus muscles associated with low back pain?. BMC Med..

[19] Rankin K.C., O’Brien L.C., Gorgey A.S. (2019). Quantification of trunk and android lean mass using dual energy x-ray absorptiometry compared to magnetic resonance imaging after spinal cord injury. J. Spinal Cord Med..

[20] Abilmona S.M., Gorgey A.S. (2018). Associations of the trunk skeletal musculature and dietary intake to biomarkers of cardiometabolic health after spinal cord injury. Clin. Physiol. Funct. Imaging.

[21] Zhang Y., Mandelli F., Mündermann A., Nüesch C., Kovacs B., Schären S., Netzer C. (2021). Association between fatty infiltration of paraspinal muscle, sagittal spinopelvic alignment and stenosis grade in patients with degenerative lumbar spinal stenosis. N. Am. Spine Soc. J..

[22] Corazzelli G., Capece M., Pizzuti V., Leonetti S., D’Elia A., Santilli M., Aloj F., Innocenzi G. (2023). Antithrombotic therapy and spinal surgery: A retrospective cohort study of 289 consecutive elderly patients with degenerative lumbar stenosis. J. Neurosurg. Spine.

[23] Mannil M., Burgstaller J.M., Thanabalasingam A., Winklhofer S., Betz M., Held U., Guggenberger R. (2018). Texture analysis of paraspinal musculature in MRI of the lumbar spine: Analysis of the lumbar stenosis outcome study (LSOS) data. Skelet. Radiol..

[24] Getzmann J.M., Ashouri H., Burgstaller J.M., Valeri F., Winklhofer S., Ulrich N.H., Guggenberger R. (2023). The effect of paraspinal fatty muscle infiltration and cumulative lumbar spine degeneration on the outcome of patients with lumbar spinal canal stenosis: Analysis of the Lumbar Stenosis Outcome Study (LSOS) data. Spine.

[25] Virk S., Sandhu M., Wright-Chisem J., Vaishnav A., Albert T., Qureshi S.A. (2021). The association between spondylolisthesis and decreased muscle health throughout the lumbar spine for patients with operative lumbar spinal stenosis. Eur. Spine J..

[26] Capece M., Corazzelli G., Pizzuti V., Leonetti S., Innocenzi G. (2023). A challenging recurrent thoracic disc herniation. Surg. Neurol. Int..

[27] Mariniello G., Corvino S., Corazzelli G., Maiuri F. (2023). Cervical epidural abscess complicated by a pharyngoesophageal perforation after anterior cervical spine surgery for subaxial spondylodiscitis. Surg. Neurol. Int..

[28] Ekşi M.Ş., Öztaş U.O., Topaloğlu F., Yeşilyurt S.C., Duymaz U.C., Osama M., Özcan-Ekşi E.E. (2024). Erector spinae could be the game changer in surgical decision-making in patients with lumbar spondylolisthesis: A cross-sectional analysis of an age-, sex-, subtype-, level-matched patients with similar spinopelvic parameters received surgical or conservative management. Eur. Spine J..

[29] Wang Z., Zhao Z., Li Z., Gao J., Li Y. (2024). Fatty infiltration in paraspinal muscles: Predicting the outcome of lumbar surgery and postoperative complications. World Neurosurg..

[30] He K., Head J., Mouchtouris N., Hines K., Shea P., Schmidt R., Hoelscher C., Stricsek G., Harrop J., Sharan A. (2020). The implications of paraspinal muscle atrophy in low back pain, thoracolumbar pathology, and clinical outcomes after spine surgery: A review of the literature. Glob. Spine J..

[31] Libda N., Fahmy H., Al Smmak A.A.E., Tantawy H.F. (2024). Correlation of Magnetic Resonance Imaging Changes of Multifidus Muscle with Other Degenerative Changes at Lumbosacral Spine in Patients with Low Back Pain. Zagazig Univ. Med. J..

[32] Assi A., Ramadan B., Karam M., Rouyer J., Mitulescu A., Campana S. (2024). Muscle fat infiltration: A narrative review of the magnetic resonance (MR)-based evaluation methods and their clinical applications. Quant. Imaging Med. Surg..

[33] Kwon H.-J., Kim C.-S., Kim S., Yoon S.H., Koh J., Kim Y.K., Choi S.-S., Shin J.-W., Kim D.-H. (2023). Association between fatty infiltration in the cervical multifidus and treatment response following cervical interlaminar epidural steroid injection. Korean J. Pain.

[34] Felippe V.G., Amaral C.A.B.d., Labronici P.J. (2022). Correlation between low back pain due to fatty degeneration and sex and age: Study by MRI. Coluna/Columna.

[35] Hildebrandt M., Fankhauser G., Meichtry A., Luomajoki H. (2017). Correlation between lumbar dysfunction and fat infiltration in lumbar multifidus muscles in patients with low back pain. BMC Musculoskelet. Disord..

[36] Vitale J., Sconfienza L.M., Galbusera F. (2024). Cross-sectional area and fat infiltration of the lumbar spine muscles in patients with back disorders: A deep learning-based big data analysis. Eur. Spine J..

